# Ideal cardiovascular health index and high-normal blood pressure in elderly people: evidence based on real-world data

**DOI:** 10.1038/s41598-024-60906-w

**Published:** 2024-05-03

**Authors:** Yongcheng Ren, Lulu Cheng, Yaoyu Song, Yuting Yang, Lin Xiang, Chaohua Wei, Tiantian Zhao, Shengnan Yu, Juan Zhang, Tiezhen Wang, Lei Yang, Xiaofang Zhang, Wei Yan, Pengfei Wang

**Affiliations:** 1 Henan Provincial Key Laboratory of Digital Medicine, Affiliated Central Hospital of Huanghuai University, Zhumadian, Henan China; 2 Institute of Health Data Management, Huanghuai University, Zhumadian, 463000 China; 3 Digital Medicine Center, Pingyu People’s Hospital, Zhumadian, 463000 Henan China

**Keywords:** Cardiovascular health index, Elderly, High-normal blood pressure, Risk factors, Disease prevention, Epidemiology

## Abstract

Limited information is available on the cardiovascular health (CVH) index and risk of high-normal blood pressure (HNBP) in elderly people. Randomized cluster sampling, multivariate logistic regression, and mediating effects analysis were used in this study analyze the relationship between CVH index and HNBP in the elderly. 1089 non-hypertensive residents aged 65 years or older completed the study. The positive rate of HNBP was 75.85% (male vs. female: 76.13% vs. 75.64%, P = 0.852); The ideal rate of CVH (ideal CVH index ≥ 5 items) was 14.51% (male vs. female: 15.91% vs. 13.46%, P = 0.256). Compared with people with 0–2 ideal CVH index, the risk of HNBP in people with 4 ideal indexes and ≥ 5 ideal indexes decreased by 50% and 63%, respectively, and their OR (95% CI) were 0.50 (0.31, 0.81) and 0.37 (0.21, 0.66), respectively. The results of the trend test showed that the risk of HNBP decreased by 32% for every increase in the ideal CVH index (trend P < 0.001) and TyG index does not play a mediating role in this relationship. That is, increasing the number of ideal CVH index may effectively reduce the risk of HNBP in elderly by one-third.

## Introduction

Abnormal blood pressure and an aging population have become one of the major public health problems facing the world^1–3^. Overall, 30.71% (≈ 323.6 million) of Chinese adult ≥ 65 years of age had high-normal blood pressure (HNBP) between 2012 and 2015^4^. The Healthy China Action (2019–2030) issued by the National Health and Wellness Committee in 2019 emphasizes that primary medical and health institutions should have the ability to provide standardized health management services for hypertension patients within their jurisdiction.

At present, the population over 65 years old in China exceeds 200 million (14.2%)^5^, which is on the edge of a moderately aging society. However, there is still a lack of accurate and effective prevention and control measures for the prevention, control, and management of abnormal blood pressure among the elderly, and we must provide real-world evidence^6^. In 2010, the American Heart Association (AHA) put forward the "AHA 2020 Impact Goal" for "improving the cardiovascular health (CVH) level of residents and reducing the risk of cardiovascular events"^7^. In this goal, AHA put forward the ideal number of seven interventional factors (smoking, body mass index (BMI), physical activity, dietary habits, blood pressure, total cholesterol (TC), and fasting blood glucose (FPG)) to distinguish different CVH levels. In different cardiovascular events, including myocardial infarction, stroke, coronary heart disease, heart failure, sudden cardiac death, angina pectoris, and all-cause death, the research on CVH has obtained specified evidence^8–11^. While studies in the natural population indicate that most factors of CVH are linked to blood pressure regulation, there is still a dearth of evidence when examining CVH as a distinct marker, particularly in relation to HNBP-related outcomes among subjects aged 65 and older.

Therefore, this study takes the elderly with non-hypertensive over 65 years old as the research object, analyzes the prevalence and association between CVH level and HNBP in this population, and provides real-world evidence based on the natural elderly population for the prevention and control of abnormal blood pressure in the elderly population.

## Methods

### Study design and sample size calculation

**Figure 1:**
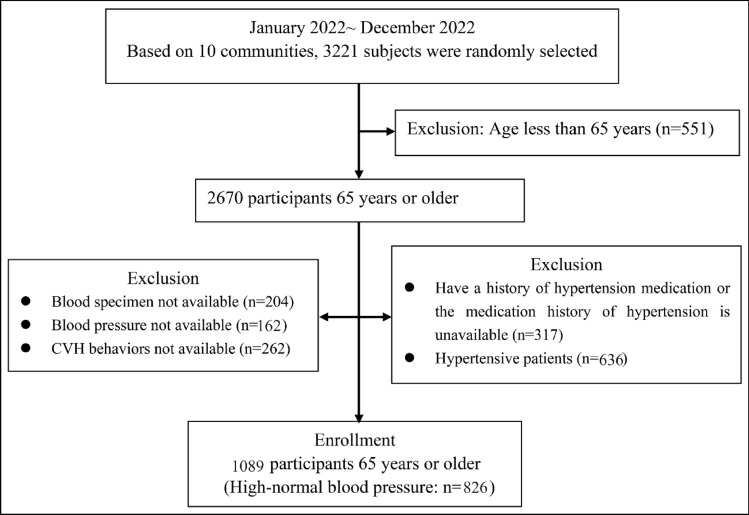
Study flow. *CVH* cardiovascular health.

From January to December 2022, using a random cluster sampling method, two streets were randomly selected from each of the 10 communities, resulting in a total of 20 streets being sampled. From each street, one household was randomly chosen to be the first household, and then every hundredth household in sequence was surveyed subsequently. This process led to the investigation of 3000 permanent residents across these 20 streets, and a total of 3221 cases were selected from the 10 communities. After excluding those aged less than 65 years and those with incomplete blood pressure, biochemical tests, CVH indicators, medication history, and hypertensive patients, a total of 1089 non-hypertensive people (826 HNBP) were included, as shown in Fig. 1**.** The Sample Size Calculations formula and Sample Size Calculations software (Mark Woodward, The George Institute International Health; Lesley Francis, MIS Consultants Pty Ltd) were applied to estimate sample content for a cross-sectional study design with outcome events as a categorical variable in epidemiological studies. Setting α = 0.05 (two-sided), β = 0.20, P_0_ = 25% for the outcome event in the non-exposed group, OR = 0.7, and a ratio of 1 for the number of people in the exposed to the non-exposed group, the required sample size was calculated to be 932. To reduce bias in the results caused by cluster sampling, the study increased the sample size by 10%, with a final sample size of 1026 cases required. This study included 1089 study subjects, which met the requirements of statistical test efficacy.

### Clinical and laboratory measurements

All data were collected by uniformly trained medical staff, and all instruments were calibrated before each use. The data collected include demographic characteristics: age, sex, occupation, nationality, education level, marital status, and medication status; Behavioral risk factors: smoking, drinking, physical activity, and dietary habits; Anthropometric indicators: height, weight, and waist circumference (WC); Blood pressure, heart rate; Biochemical indicators: TC, triglyceride (TG), low-density lipoprotein (LDL-C), high-density lipoprotein (HDL-C), and FPG. A questionnaire was used to collect information on demographic characteristics and behavioral factors. A clinical examination was used to collect anthropometric indicators. Overnight fasting blood samples were collected and stored at − 20 °C for measuring biochemical indicators by use of an automatic biochemical analyzer (Hitachi 7060, Tokyo). Based on the policy statement and call to action from the world hypertension league^12^, blood pressure was measured on the bare upper arm after at least 5 min of rest by an electronic sphygmomanometer (Omron, HEM‐770AFuzzy, Kyoto). Appropriate cuff size was chosen by the medical experts and was measured at least twice for accurate blood pressure value and the average value of each blood pressure was registered in the health screening data. Antihypertensive medication use was defined as taking medication regularly for 1 month or more.

### Definition of key variables

Based on the standard of the National Manual for Prevention and Treatment of Primary Hypertension (2020 Edition)^13^, the diagnostic criteria of HNBP are in-office sitting blood pressure, systolic blood pressure (SBP) 120–139 mmHg and/or diastolic blood pressure (DBP) 80–89 mmHg under non-drug treatment. Hypertension is diagnosed as in-office sitting blood pressure under non-pharmacological treatment SBP ≥ 140 mmHg and/or DBP ≥ 90 mmHg or taking antihypertensive medication. Antihypertensive medication use was defined as taking the medication regularly for 1 month or more. Ideal CVH behavior is defined as (1) Ideal Smoking: the number of smokers in the subjects was less than 100 as of the survey date; (2) Ideal BMI: < 24 kg/m^2^; (3) Ideal diet: reasonable mix of meat and vegetables, no salt and oil; (4) Ideal physical activity: light physical activity for more than 30 min and/or moderate physical activity for more than 20 min and/or heavy physical activity for more than 10 min every day; (5) Ideal TC level: < 5.18 mmol/L; (6) Ideal FPG level: < 5.6 mmol/L. The outcome event of this study is HNBP, so the ideal CVH index in this study does not include blood pressure. Triglyceride glycemic index (TyG): One of the evaluation indexes of insulin resistance level, the calculation formula is: TyG = Ln (TG × 88. 55 × FPG × 18/2) (TG and FPG units are mmol/L).

### Statistical analysis

Classified variables were described by percentage and analyzed by χ^2^ test. Continuous variables were described by median (IQR) and analyzed by non-parametric test. A multivariate Logistic regression model was used to analyze the association between ideal CVH levels and HNBP, and described by OR and 95% CI (adjusted for age, gender, education, marital status, heart rate, WC, HDL-C, LDL-C, TG). The PROCESS procedure was used to analyze the mediating effect between TyG-mediated CVH level and HNBP with CVH level as the independent variable (X), blood pressure as the dependent variable (Y), and TyG as the mediating variable (M). The mediating effect and 95% CI were obtained using a bias-corrected non-parametric percentile bootstrap method with 5000 random sampling times. SPSS 23.0 statistical software was used for all the analyses. Bilateral test level α = 0.05.

### Ethics approval and consent to participate

This study was conducted according to the guidelines laid down in the Declaration of Helsinki and all procedures involving human subjects were approved by the Ethics Committee of Huanghuai University. Written informed consent was obtained from all enrolled patients.

## Results

### Basic characteristics of subjects

A total of 1089 subjects were included in the study (Table 1), with an average age of 69.17 years, including 465 males (42.70%) and 624 females (57.30%). The positive rate of HNBP was 75.85%, and there was no significant difference between males and females (male vs. female: 76.13% vs. 75.64%, P = 0.852). The ideal rate of CVH (ideal CVH index ≥ 5 items) was 14.51% (male vs. female: 15.91% vs. 13.46%, P = 0.256). The levels of TyG, TC, TG, HDL-C, and LDL-C were significantly different between men and women (P < 0.001). Comparison of basic characteristics between normal blood pressure and HNBP in elderly people (Table 2), there was no statistically significant difference in the comparison of other indicators except for age, the ideal rate of CVH, and BMI.

**Table 1:** Basic characteristics of subjects.

Characteristic	All (n = 1089)	Male (n = 465)	Female (n = 624)	P value *
Age (median (IQR), years)	69.17 (66.92, 73.85)	69.49 (67.02, 73.69)	68.94 (66.76, 73.99)	0.525
High school and above (%)	214 (19.65)	119 (25.59)	95 (15.22)	< 0.001
Married (%)	1031 (94.67)	452 (97.2)	579 (92.79)	0.001
HNBP (%)	826 (75.85)	354 (76.13)	472 (75.64)	0.852
CVHR (%)	158 (14.51)	74 (15.91)	84 (13.46)	0.256
HR (median (IQR), times/min)	74 (68, 78)	74 (69, 78)	74 (68, 79)	0.801
BMI (median (IQR), kg/m^2^)	24.14 (22.32, 26.04)	23.94 (22.14, 25.91)	24.27 (22.51, 26.14)	0.114
SBP (median (IQR), mmHg)	124 (118, 130)	125 (118, 130)	124 (118, 130)	0.704
DBP (median (IQR), mmHg)	75 (70, 80)	75 (70, 80)	74 (68, 80)	0.014
FPG (median (IQR), mmol/L)	5.19 (4.59, 5.83)	5.18 (4.57, 5.84)	5.20 (4.60, 5.83)	0.994
TyG (median (IQR))	8.74 (8.35, 9.17)	8.62 (8.23, 9.04)	8.84 (8.49, 9.25)	< 0.001
TC (median (IQR), mmol/L)	5.06 (4.32, 5.86)	4.79 (4.12, 5.57)	5.30 (4.51, 6.05)	< 0.001
TG (median (IQR), mmol/L)	1.51 (1.05, 2.16)	1.31 (0.92, 1.87)	1.66 (1.18, 2.30)	< 0.001
HDL-C (median (IQR), mmol/L)	1.59 (1.33, 1.91)	1.53 (1.26, 1.82)	1.67 (1.39, 1.99)	< 0.001
LDL-C (median (IQR), mmol/L)	3.03 (2.51, 3.67)	2.89 (2.38, 3.47)	3.16 (2.57, 3.79)	< 0.001

*HNBP* high-normal blood pressure, *CVHR* The ideal rate of cardiovascular health behavior, that is, the proportion of ideal number exceeding 5, *HR* Heart rate, *BMI* Body mass index, *SBP* Systolic blood pressure, *DBP* Diastolic pressure, *FPG* Fasting blood glucose, *TyG index* triglyceride glycemic index, *TC* Total cholesterol, *TG* Triglyceride, *HDL-C* High density lipoprotein cholesterol, *LDL-C* Low density lipoprotein cholesterol.

*Male vs. female.

### The relationship between ideal CVH indicators and HNBP

After adjusting for age, gender, education, marital status, heart rate, WC, HDL-C, LDL-C, and TG, compared with the population with 0–2 ideal CVH indicators, the risk of HNBP in the elderly with 4 and ≥ 5 ideal indicators decreased by 50% and 63% respectively, and their OR (95% CI) was 0.50 (0.31, 0.81) and 0.37 (0.21, 0.66) respectively (Table 3). The results of the trend test showed that for every increase of the ideal CVH index in the elderly population, the current risk of adjusted HNBP could be reduced by 32%, and its OR (95% CI) was 0.68 (0.57, 0.81), with trend P < 0.001 (Table 3).

**Table 2:** Comparison of basic characteristics between normal blood pressure and HNBP in elderly people.

Characteristic	Normal blood pressure (n = 263)	HBPN (n = 826)	*P value*
Age (median (IQR), years)	68.82 (66.55, 72.96)	69.32 (67.01, 74.13)	0.038
male (%)	111 (57.79)	354 (42.86)	0.852
High school and above (%)	45 (17.11)	169 (20.46)	0.234
Married (%)	250 (95.1)	781 (94.55)	0.741
CVHR (%)	53 (20.15)	105 (12.71)	0.003
HR (median (IQR), times/min)	73 (67, 78)	74 (69, 78)	0.071
BMI (median (IQR), kg/m^2^)	23.24 (21.63, 25.72)	24.31 (22.66, 26.17)	< 0.001
SBP (median (IQR), mmHg)	110 (103, 115)	129 (121, 132)	< 0.001
DBP (median (IQR), mmHg)	68 (62, 70)	78 (70, 80)	< 0.001
FPG (median (IQR), mmol/L)	5.11 (4.61, 5.65)	5.21 (4.95, 5.90)	0.102
TyG (median (IQR))	8.80 (8.29, 9.17)	8.72 (8.36, 9.18)	0.649
TC (median (IQR), mmol/L)	4.94 (4.28, 5.71)	5.11 (4.32, 5.89)	0.237
TG (median (IQR), mmol/L)	1.57 (1.03, 2.25)	1.48 (1.05, 2.13)	0.400
HDL-C (median (IQR), mmol/L)	1.57 (133, 1.87)	1.60 (1.33, 1.93)	0.209
LDL-C (median (IQR), mmol/L)	2.96 (2.48, 3.56)	3.07 (2.52, 3.71)	0.121

Classification variables are described by the number of cases (%).

*HNBP* high-normal blood pressure, *CVHR* The ideal rate of cardiovascular health behavior, that is, the proportion of ideal number exceeding 5, *HR* Heart rate, *BMI* Body mass index, *SBP* Systolic blood pressure, *DBP* Diastolic pressure, *FPG* Fasting blood glucose, *TyG index* triglyceride glycemic index, *TC* Total cholesterol, *TG* Triglyceride, *HDL-C* High density lipoprotein cholesterol, *LDL-C* Low density lipoprotein cholesterol.

### TyG-mediated mediating effects

Taking people with ≥ 5 ideal CVH indexes as a reference, the total effect (path c) and direct effect (path c') of ideal CVH index and HNBP are statistically significant, and their adjusted OR (95% CI) are 1.75 (1.19–2.59) and 1.62 (1.08–2.41). The indirect effect (path ab #, mediating effect) of ideal CVH index and HNBP has no statistical significance, and the adjusted OR (95% CI) is 1.07 (0.98–1.19). That is, TyG index does not play a mediating role in this relationship (Table 4).

**Table 3:**
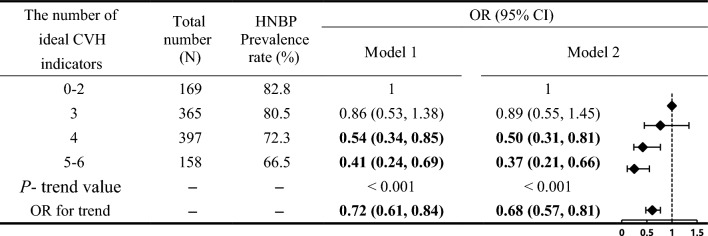
Relationship between ideal CVH indexes and HNBP in the elderly.

*HNBP* high-normal blood pressure, *CVH* Cardiovascular health, *OR* odds ratio, *CI* Confidence interval.

Model 1: Unadjusted; Model 2: Adjust age, gender, education level, marital status, heart rate, high density lipoprotein, low density lipoprotein, and triglyceride.

**Table 4:** Mediating effect of TyG on the relationship between ideal CVH index and HNBP.

Paths in the mediation model	Beta value (95% CI) *	OR value (95% CI) *	*P*
Total effect-path c	0.56 (0.17–0.95)	1.75 (1.19–2.59)	0.005
Direct effect-path c'	0.48 (0.08–0.88)	1.62 (1.08–2.41)	0.018
Path a	0.19 (0.14–0.25)	–	< 0.001
Path b	0.39 (-0.08–0.87)	1.48 (0.92–2.39)	0.106
Mediating effect-path ab #	0.07 (-0.02–0.17)	1.07 (0.98–1.19)	> 0.050

*HNBP* high-normal blood pressure, *TyG* Triglyceride glycemic index, *CVH* Cardiovascular health.

*Adjusting factors include age, gender, educational level, marital status, heart rate, high-density lipoprotein, low-density lipoprotein, and triglyceride.

## Discussion

This study shows that the detection rate of HNBP in the non-hypertensive elderly population is as high as 75.85%. The ideal rate of CVH is 14.51%, and only 13.46% in women. Ideal CVH behavior is a protective factor for the occurrence of HNBP. Based on real-world data, every increase of the ideal CVH index may effectively reduce the current risk of HNBP in elderly people by 32% (trend *P* < 0.001).

According to the data released by the National Cardiovascular Center in 2022, the overall detection rate of HNBP among adults in China is 41.3%^14^, which is far lower than the detection rate of 75.85% of HNBP among the elderly. The results suggest that the high-risk groups of hypertension are mainly concentrated among the elderly over 65 years old. Existing studies have shown that about half of vascular deaths can be attributed to SBP > 120 mmHg^15^. In addition, the research based on The Prospective Studies Collaboration (PSC) shows that the death risk of stroke, ischemic heart disease, or other vascular diseases have doubled since 115/75 mmHg for every 20 mmHg increase of SBP and 10 mmHg increase of DBP. Based on China Kadoorie Biobank (CKB), for every 10 mmHg decrease in SBP, the death risk of ischemic stroke, coronary heart disease and intracranial hemorrhage can be reduced by 23%, 23% and 40% respectively^16^. Therefore, early prevention, early detection, early control, and early management of HNBP in the elderly have become one of the important core strategies to curb the epidemic of cardiovascular and cerebrovascular diseases.

A meta-analysis of 88 studies in the world in 2018 showed that the ideal CVH rate (ideal CVH index ≥ 5 items) in the population was 19.6% ^17^, which was much higher than the ideal CVH rate of 14.51% in the elderly population. The results suggested that the elderly non-hypertensive population paid less attention to the ideal cardiovascular behavior-related indicators. Studies have shown that with the increase in CVH level, cardiovascular events, all-cause mortality, and vascular events in the elderly decreased^8,10,18^. Our study shows that compared with the population with 0–2 ideal CVH indicators, the risk of HNBP in the population with 4 ideal indicators and ≥ 5 ideal indicators is reduced by 50% and 63%, respectively. At the same time, the trend test showed that every 1 increase in the ideal CVH index number could reduce the risk of HNBP by 32%, P < 0.001. However, deal CVH rate decreases with age at population-level, and it is very low for the elderly. From a preventive perspective, we suggest implementing interventions from earlier life stages to achieve the primordial prevention strategy of ideal CVH.

This study has several limitations. Firstly, the study population is all aged 65 years or older in China. Although the shreds of evidence are more relevant in guiding health interventions for the elderly, there was a progressive increase in cuff pulse pressure underestimation of invasive aortic pulse pressure with increasing decades of age^19^, thus the extrapolation of the findings to other ages or geographical groups and the interpretation of causal associations will be somewhat limited. Secondly, 1089 participants were included in this study, and although the sample size can meet the statistical efficacy of this study, the stability of its findings still needs to be verified by a large sample of studies. The findings of this study can be used as an a priori hypothesis for further exploration in subsequent studies. Thirdly, due to age or other physical reasons, some people could not be included in this study because of the absence of critical variables, although the measures of expanded sample size in the design phase and multivariate adjustment in the statistical analysis phase were applied, which might still partially bias the study findings. Finally, sleep was not included in the study, which was included in the updated definition of the AHA^20^, the guidance of the conclusions of this study may be limited in its specificity.

In conclusion, based on real-world evidence, the detection rate of HNBP in elderly people aged 65 years or older is extremely high. Enhancing the number of ideal CVH indexes may effectively decrease the risk of HNBP in the elderly by one-third.

## Data Availability

The datasets used and/or analysed during the current study available from the corresponding author on reasonable request.
